# The complete mitochondrial genome of the firefly, *Abscondita anceyi* (Olivier) (Coleoptera: Lampyridae)

**DOI:** 10.1080/23802359.2018.1456373

**Published:** 2018-04-02

**Authors:** Jinfeng Hu, Xinhua Fu

**Affiliations:** a Fujian Key laboratory for Monitoring and Integrated Management of Crop Pest, Institute of Plant Protection, Fujian Academy of Agricultural Science, Fuzhou, Fujian, China;; b Hubei Insect Resources Utilization and Sustainable Pest Management Key Laboratory, College of Plant Science and Technology, Huazhong Agricultural University, Wuhan, Hubei, China;; c Firefly Conservation Research Centre, Wuhan, Hubei, China

**Keywords:** *Abscondita anceyi*, firefly, Lampyridae, mitochondrial genome

## Abstract

We report the complete mitochondrial genome of firefly, *Abscondita anceyi* (Olivier). The circular genome of 16,519 bp has a base composition of A (43.81%), C (11.80%), G (8.35%), and T (36.03%). Similar to other Metazoa, our sequence contains 13 protein-coding genes, 22 transfer RNA genes, two ribosomal RNA genes, and a non-coding AT-rich region. We sequenced the mitochondrial genome of fireflies to analyse phylogenetic relationships and deduce the evolution of their flashing signals.

## Introduction

Fireflies are well known as luminous beetles, and there are approximately 100 genera and 2000 species of fireflies distributed around the world (McDermott 1964, 1966; Minami 1983). Through morphological analysis, the genus *Abscondita* was erected in the Luciolinae in 2013 (Ballantyne et al. 2013). In this genus, *Abscondita anceyi* has specifically two kind of flash pattern (slow pattern, fast pattern), distinguished from other species by its larger size, pale abdomen and black elytral apices (Ballantyne et al. 2013; Fu 2014).

Mitochondrial genome sequences are essential to a comprehensive understanding of the evolution of Lampyridae and other luminescent beetles (Ermakov et al. 2006). Here, we elucidate the mtDNA genome of *Abs. anceyi*.

These fireflies used in this study were collected from Yichang City, Hubei province, China (30°84′N, 111°08′E), and were stored in Natural History Museum, Huazhong Agricultural University, Wuhan, Hubei, China (accession no. AB2014062001).

Specific primers were designed based on these conserved regions sequences. The PCR reaction was carried out with LA Taq polymerase for 35 cycles at 94 °C for 30 s, and annealed at 50 °C for 30 s, followed by extension at 72 °C for 1 min per 1 kb. Sequences were assembled using the software DNAstar v7.1 (DNAstar, Madison, WI) and adjusted manually to generate the complete sequence of mitochondrial DNA.

The complete mitochondrial genome sequence of *Abs. anceyi* (GenBank: MH020192) has 16,519 bp and has a base composition of A (43.81%), C (11.80%), G (8.35%), T (36.03%), and GC content was 20.15%. Similar to other Metazoa, our sequence contains 13 protein-coding genes, 22 transfer RNA genes, two ribosomal RNA genes and a non-coding AT-rich region, which represents a typical insect mitochondrial genome (Wolstenholme 1992). The open frames of the 13 protein-coding genes were inferred from three other fireflies: *Aquatica leii*, *Luciola substriata* (recently identified as *Sclerotia flavida* by Ballantyne et al. 2016), and *Pyrocoelia rufa* (Lee et al. 2004; Jiao et al. 2015; Mu et al. 2016). All 13 PCGs initiated with ATN (ATT, ATA, and ATG) codon. Among those genes, five PCGs initiate from ATG (COII, ATP6, COIII, ND4, CTYB), and five PCGs initiate from ATT (COI, ND3, ND5, ND4L, ND1), and three PCGs start with ATA (ND2, ATP8, ND6). In addition, an incomplete terminal codon namely single T was found in five PGGs (COI, COII, COIII, ND5, ND4), In case of other eight PCGs, TAA (ND2, ATP8, ATP6, ND4L, ND6) or TAG (ND3, CTYB, ND1) was used.

**Figure 1.**
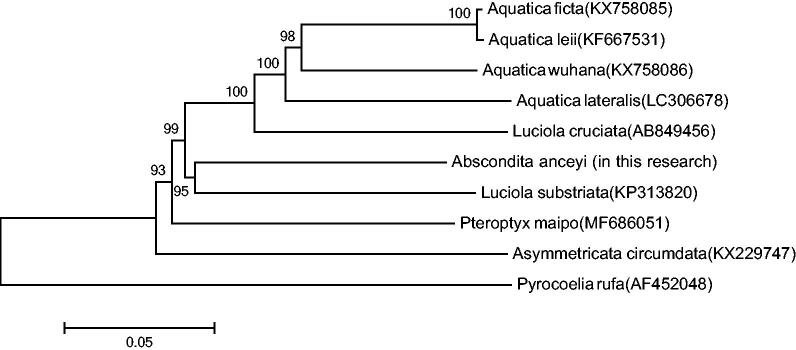
Molecular phylogeny of *Abs. anceyi* and nine other firefly species based on the complete mitochondrial genome. The complete mitochondrial genome was downloaded from GenBank and the phylogenic tree was constructed by neighbour-joining method with 1000 bootstrap replicates. MtDNA accession numbers used for tree construction are as follows: *Pteroptyx maipo* (MF686051) *Aquatica ficta* (KX758085), *Pyrocoelia rufa* (AF452048), *Aquatica leii* (KF667531), *Aquatica wuhana* (KX758086), *Luciola cruciata* (AB849456), *Asymmetricata circumdata* (KX229747), *Aquatica lateralis* (LC306678), and *Luciola substriata* (recently identified as *Sclerotia flavida* by Ballantyne et al. 2016) (KP313820).

The phylogenetic tree among the five species based on mitochondrial genome sequences was aligned in MEGA 5 (Phoenix, AZ) (with 1000 bootstrap replicates) to construct a Neighbour-Joining tree (Figure 1). The result shows *Abs. anceyi* is most closely related to *L. substriata* (recently identified as *Sclerotia flavida* by Ballantyne et al. 2016), which belongs to an entirely different genus in the Lampyridae.

In conclusion, the complete mitochondrial genome sequence of *Abs. anceyi* provides an important molecular framework for further phylogenetic analyses of fireflies.
